# Supplementary material for: Elevated polyadenylated histone gene transcription in human cancer
Source: bioRxiv. 2026 Oct 20:2026.10.14.758961. Preprint. [Version 1] doi: 10.64898/2026.10.14.758961 (PMC13738363; doi:10.64898/2026.10.14.758961)
Supplement: Supplementary file 1 [file NIHPP2026.10.14.758961v1-supplement-1.pdf]

bioRxiv preprint doi: https://doi.org/10.64898/2026.10.14.758961; this version posted October 20, 2026. The copyright holder for this preprint (which was not certified by peer review) is the author/funder, who has granted bioRxiv a license to display the preprint in perpetuity. It is made available under a CC-BY 4.0 International license.

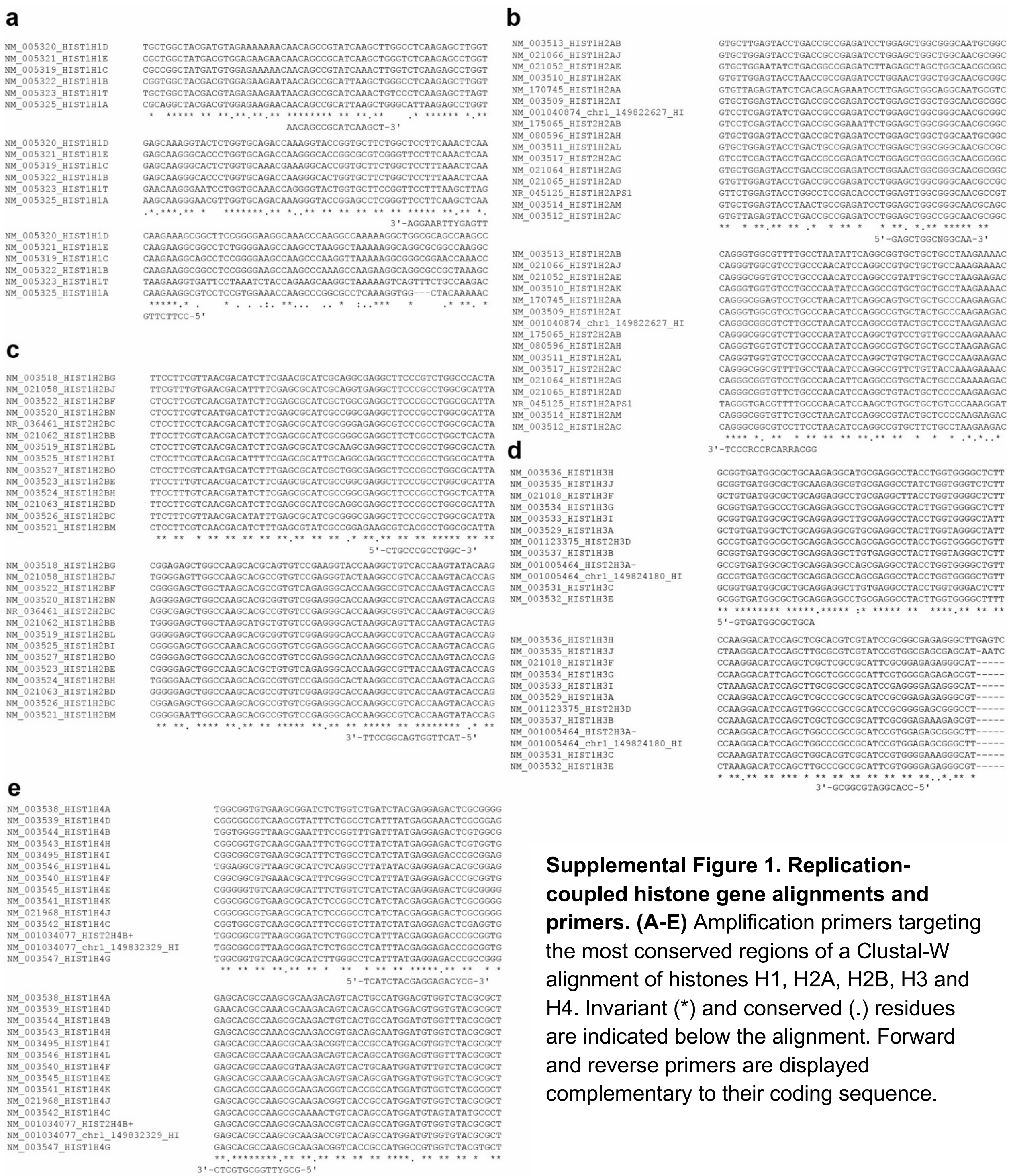

Supplemental Figure 1. Replication-coupled histone gene alignments and primers. (A-E) Amplification primers targeting the most conserved regions of a Clustal-W alignment of histones H1, H2A, H2B, H3 and H4. Invariant (*) and conserved (.) residues are indicated below the alignment. Forward and reverse primers are displayed complementary to their coding sequence.

bioRxiv preprint doi: https://doi.org/10.64898/2026.10.14.758961; this version posted October 20, 2026. The copyright holder for this preprint (which was not certified by peer review) is the author/funder, who has granted bioRxiv a license to display the preprint in perpetuity. It is made available under a CC-BY 4.0 International license.

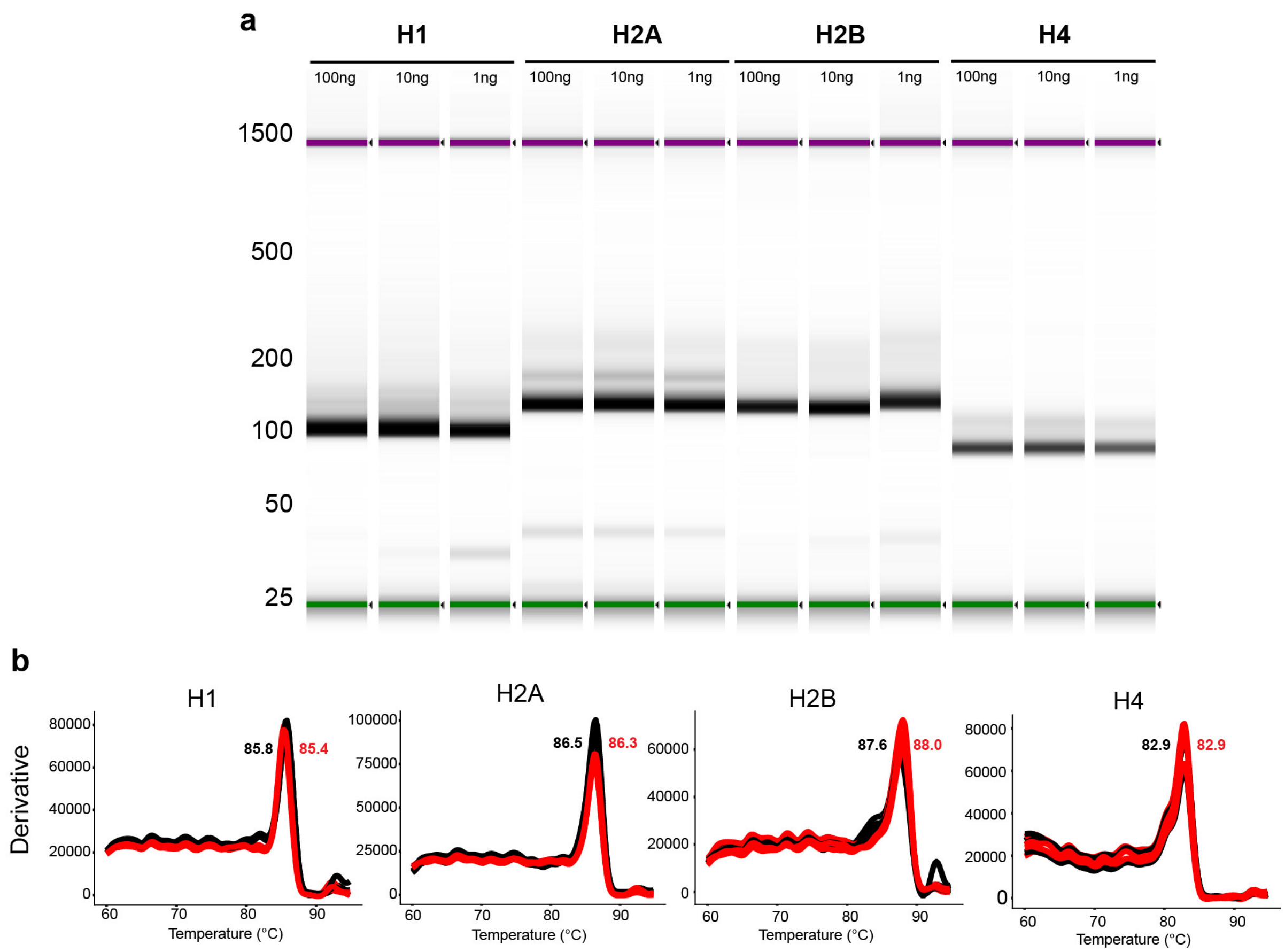

c

Primer	Description	% Identity	RefSeq
H1	Homo sapiens H4 clustered histone 11 (H4C11), mRNA	100%	NM_021968.4
H2A	Homo sapiens H2A clustered histone 19 (H2AC19), mRNA	99.27%	NM_001040874.1
H2B	Homo sapiens H2B clustered histone 12 (H2BC12), transcript variant 1, mRNA	99.25%	NM_001312653.2
H4	Homo sapiens H4 clustered histone 11 (H4C11), mRNA	100%	NM_021968.4

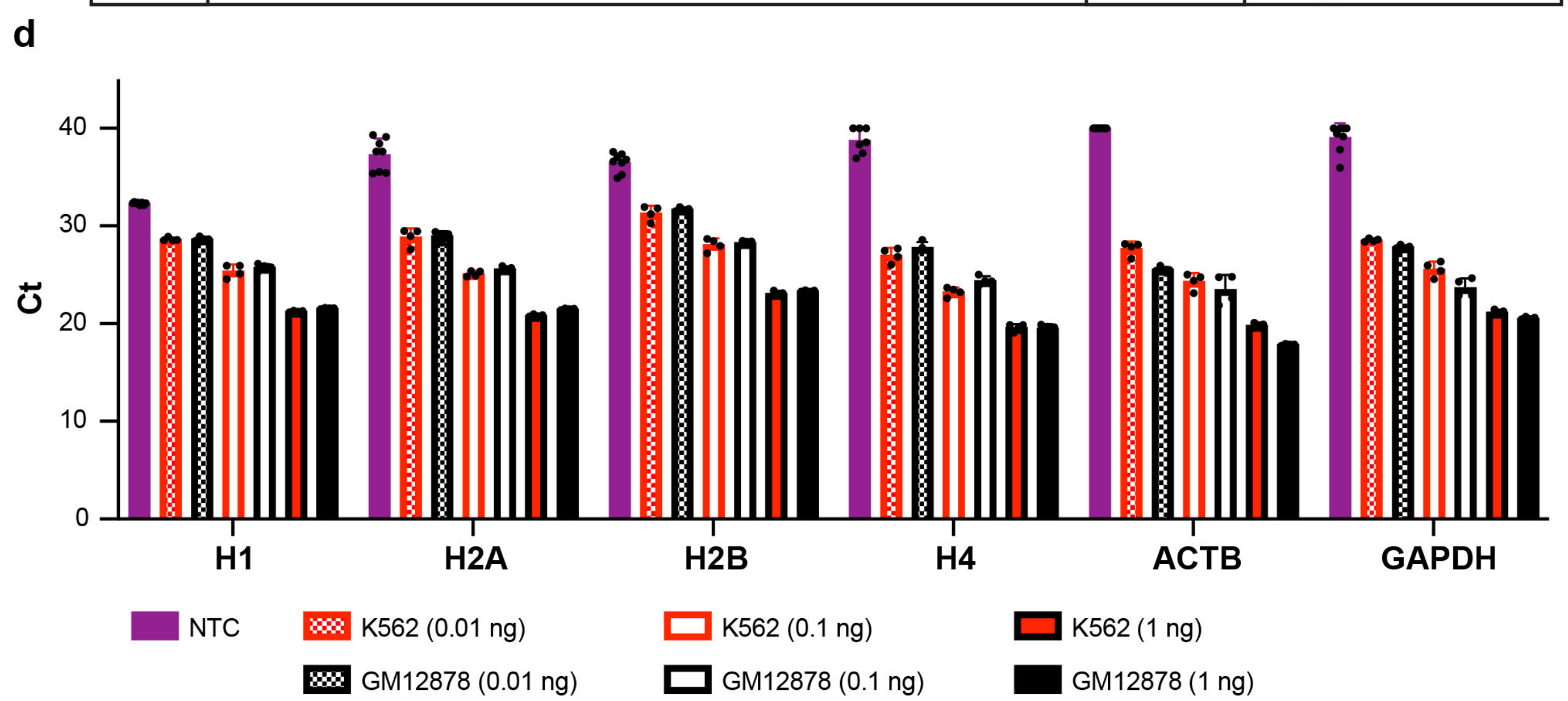

bioRxiv preprint doi: https://doi.org/10.64898/2026.10.14.758961; this version posted October 20, 2026. The copyright holder for this preprint (which was not certified by peer review) is the author/funder, who has granted bioRxiv a license to display the preprint in perpetuity. It is made available under a CC-BY 4.0 International license.

Supplemental Figure 2. Validation of degenerate histone gene primers. (A). Automated capillary electrophoresis of PCR-amplified cDNA generated using degenerate histone gene primers. Lanes are grouped by target with each group representing a 100 ng, 10 ng, and 1 ng cDNA input series. **(B)** Derivative melt curves are shown for each primer set for 1 ng input cDNA (red = K562, black = GM12878). The melting temperature for each cell line was determined as the temperature at the maximum derivative signal and is annotated in each panel. **(C)** Consensus sequences were generated from amplification-free, single-molecule long-read sequencing of each amplicon. Table showing top BLASTn hit for each consensus read (NCBI core_nt, Homo sapiens). **(D)** Ct values for K562 and GM12878 cDNA (0.01 ng – 1ng) are compared to no template controls (NTC), where each point represents an individual technical replicate (n = 4-8), bars indicate mean with SD, and undetectable samples are assigned a Ct of 40.

bioRxiv preprint doi: https://doi.org/10.64898/2026.10.14.758961; this version posted October 20, 2026. The copyright holder for this preprint (which was not certified by peer review) is the author/funder, who has granted bioRxiv a license to display the preprint in perpetuity. It is made available under a CC-BY 4.0 International license.

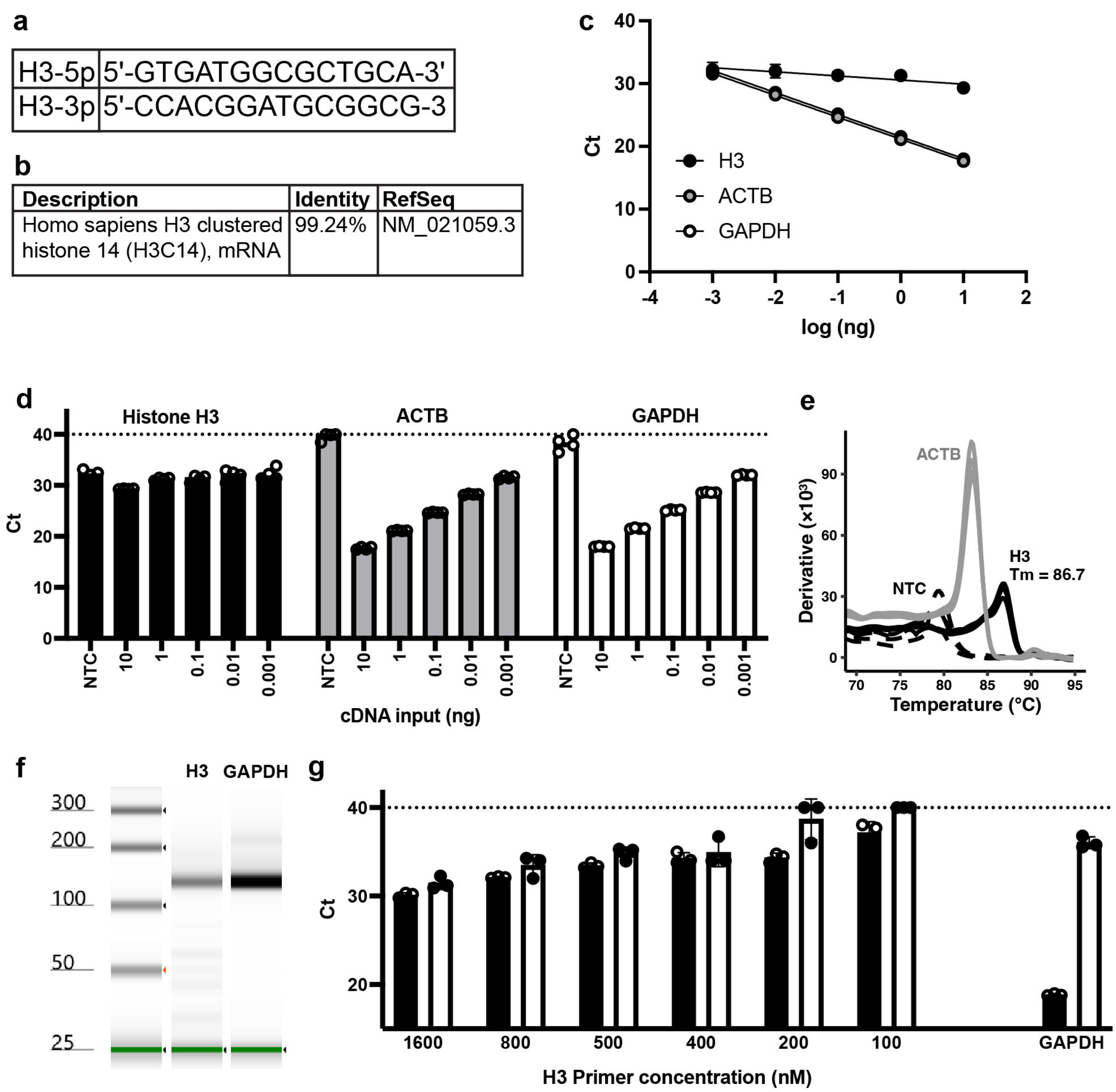

Supplemental Figure 3. Characterization of histone H3 Primers. (A). Degenerate primers targeting the most conserved regions of histone H3. **(B)** Long-read sequencing of amplicons generated from K562 RNA by random hexamer priming and PCR using degenerate histone H3 primers identified H3_1 as the top BLASTn hit (NCBI core_nt, Homo sapiens). **(C,D)** qPCR was performed using serial dilutions (0.001–10 ng) of cDNA prepared by random hexamer-primed reverse transcription of K562 RNA. (C) Standard curves for histone H3 (black), ACTB (gray), and GAPDH (white) were generated from these reactions. Data points represent the mean of four technical replicates; error bars indicate SD, and solid lines represent linear regression fits for each primer set. (D) Ct values from reactions in (C) were plotted alongside no-template controls (NTCs). Each point represents an individual technical replicate (n = 4), and bars indicate the mean ± SD (undetectable reactions were assigned Ct of 40 shown by dotted line). **(E)** Derivative melt curve of reactions from (C).

bioRxiv preprint doi: https://doi.org/10.64898/2026.10.14.758961; this version posted October 20, 2026. The copyright holder for this preprint (which was not certified by peer review) is the author/funder, who has granted bioRxiv a license to display the preprint in perpetuity. It is made available under a CC-BY 4.0 International license.

Traces are shown for histone H3 at 10 ng (solid black), no-template control (dashed), and ACTB (grey). **(F)** Automated capillary electrophoresis of PCR-amplified products from histone H3 and GAPDH primers from 10ng reactions in (C). **(G)** Ct values across histone H3 primer titration. qPCR performed on 5ng of random hexamer primed cDNA (template, black) using GAPDH and histone H3 primers (100 to 1600nM) compared to no-template controls (NTC, white). Bars indicate the mean of three technical replicates, error bars indicate SD, and individual replicates are plotted as points. No primer concentration suppressed background amplification with reliable detection of histone H3 product.

bioRxiv preprint doi: https://doi.org/10.64898/2026.10.14.758961; this version posted October 20, 2026. The copyright holder for this preprint (which was not certified by peer review) is the author/funder, who has granted bioRxiv a license to display the preprint in perpetuity. It is made available under a CC-BY 4.0 International license.

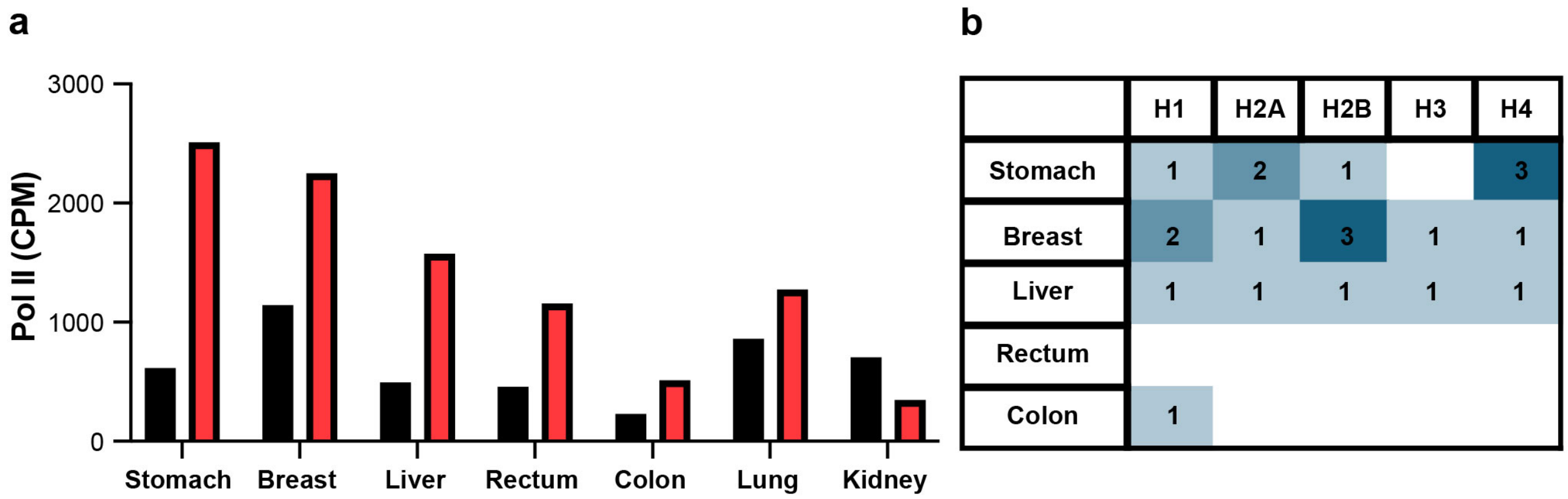

Supplemental Figure 4. Individual histone genes are relatively decreased in tumors exhibiting cancer-associated hypertranscription and increased RNAPII occupancy at histone locus clusters. **(A)** RNAPII Signal at histone genes calculated as in (Fig. 3B) across multiple tumors and matched normal samples (black = normal, red = tumor) from our previous study (1). **(B)** Presence–absence matrix summarizing histone gene downregulation in samples that demonstrated genome-wide RNAPII hypertranscription. Each cell shows the number of histone genes decreased in the tumor sample relative to its matched normal.

bioRxiv preprint doi: https://doi.org/10.64898/2026.10.14.758961; this version posted October 20, 2026. The copyright holder for this preprint (which was not certified by peer review) is the author/funder, who has granted bioRxiv a license to display the preprint in perpetuity. It is made available under a CC-BY 4.0 International license.

a

Histone Gene	GM12878 poly(A)	K562 poly(A)	poly(A): K562-GM12878	GM12878 total RNA	K562 total RNA	total: K562-GM12878
H1-2	141.9	928.93	787.03	733.71	1577.73	844
H2BC12	97.15	823.71	726.56	636.03	978.84	342.8
H2BC11	17.3	185.73	168.43	326.8	491.46	164.7
H2AC6	15.23	104.63	89.4	55.81	132.73	76.92
H3C10	3.48	53.7	50.22	194.18	153.23	-41
H4C14	23.22	72.96	49.74	944.47	1020.31	75.84
H2BC4	15.41	63.47	48.06	472.09	425.83	-46.3
H2BC5	15.1	52.21	37.11	375.01	340.81	-34.2
H2AC8	14.76	50.4	35.64	452.55	428.07	-24.5
H2AC13	4.98	36.33	31.35	622.32	687.56	65.24
H4C8	19.58	49.53	29.95	713.36	287.81	-426
H2BC8	4.06	30.88	26.82	94.2	151.24	57.04
H4C15	5.48	29.46	23.98	8.04	29.39	21.35
H2AC11	2.5	24.74	22.24	117.06	223.08	106
H2BC17	7.21	28.22	21.01	214.44	361.15	146.7
H2BC21	2.25	22.99	20.74	51.69	102.76	51.07
H2BC15	4.04	20.66	16.62	78.16	120.16	42
H2AC17	12.7	24.61	11.91	390.44	563.06	172.6
H4C5	8.24	17	8.76	118.67	125.42	6.75
H2AC15	1.21	9.58	8.37	85.49	176.07	90.58
H1-4	6.21	13.48	7.27	436	591.31	155.3
H2BC13	6.87	12.87	6	326.09	349.74	23.65
H4C2	7.71	12.51	4.8	419.27	382.68	-36.6
H4C9	1.98	5.71	3.73	67.77	49.49	-18.3
H2BC7	5.89	9.57	3.68	125.23	101.64	-23.6
H2BC18	2.53	5.64	3.11	194.42	178.18	-16.2
H2BC19P	4.16	7.21	3.05	3.05	6.1	3.05
H3C13	1.37	3.9	2.53	318.82	430.64	111.8
H3C14	0.77	3.09	2.32	127.13	175.72	48.59
H3C15	0.77	3.09	2.32	127.13	174.07	46.94
H2BC20P	1.65	3.86	2.21	1.34	2.31	0.97
H2AC12	7.8	9.53	1.73	334.83	650.43	315.6
H4C4	2.28	4	1.72	727.23	326.17	-401
H2AC1	0.01	0.94	0.93	0.01	10.32	10.31
H4C10P	0.09	0.79	0.7	0.06	0.01	-0.05
H2BC16P	0.21	0.74	0.53	0.18	0.37	0.19
H1-12P	0.08	0.5	0.42	0.06	0.05	-0.01
H1-6	0.06	0.41	0.35	0.2	1	0.8
H3C9P	0.35	0.64	0.29	0.3	0.23	-0.07
H2AC21	0.67	0.92	0.25	79.27	108.75	29.48
H2BC1	0.01	0.19	0.18	0.04	18.16	18.12
H2AC5P	0.06	0.13	0.07	2.45	0.04	-2.41
H3C5P	0	0.05	0.05	0.03	0.04	0.01
H3-7	0	0.03	0.03	0	0.01	0.01
H4C7	0	0.02	0.02	0.03	0.03	0
H2BC2P	0	0.01	0.01	0	0.06	0.06
H2AC3P	0	0.01	0.01	0	0	0
H2AC9P	0	0	0	0	0	0
H2AC2P	0.01	0	-0.01	0	0	0
H2AC20	3.47	3.38	-0.09	162	474.63	312.6
H2AC10P	0.36	0	-0.36	0.72	0	-0.72
H2AC4	0.99	0.61	-0.38	186.3	220.3	34
H1-1	0.45	0.04	-0.41	2.21	0.08	-2.13
H2BC9	4.07	3.65	-0.42	87.94	12.06	-75.9
H3C4	1.64	0.96	-0.68	91.12	20.77	-70.4
H3C6	0.96	0.16	-0.8	4.36	0.08	-4.28
H2BC3	1.01	0.17	-0.84	133.73	0.41	-133
H3C1	1.37	0.38	-0.99	73.78	17.76	-56
H3C8	1.42	0	-1.42	88.12	0.02	-88.1
H4C6	1.6	0	-1.6	246.36	0.19	-246
H3C7	2.59	0.77	-1.82	446.47	94.31	-352
H4C1	5.81	3.97	-1.84	96.61	72.96	-23.7
H2BC10	1.93	0	-1.93	315.99	0.15	-316
H4C13	2.27	0	-2.27	164.89	0.09	-165
H3C11	2.53	0	-2.53	153.18	0.2	-153
H4C11	16.39	13.64	-2.75	1614.93	1741.85	126.9
H4C12	6.6	3.74	-2.86	2.35	82.91	80.56
H1-3	5.74	2.41	-3.33	261.9	128.81	-133
H2BC6	5.22	0.83	-4.39	169.18	0.95	-168
H2AC7	11.75	7.35	-4.4	68.69	318.26	249.6
H1-5	5.95	1.12	-4.83	522.82	164.67	-358
H2AC16	12.76	4.7	-8.06	249.74	126.92	-123
H2BC14	8.61	0.08	-8.53	299.93	0.17	-300
H2AC14	10.8	0	-10.8	470.56	0.25	-470
H3C12	17.04	2.11	-14.93	237.79	137.93	-99.9
H4C3	1705.58	1371.16	-334.42	464.7	519.55	54.85

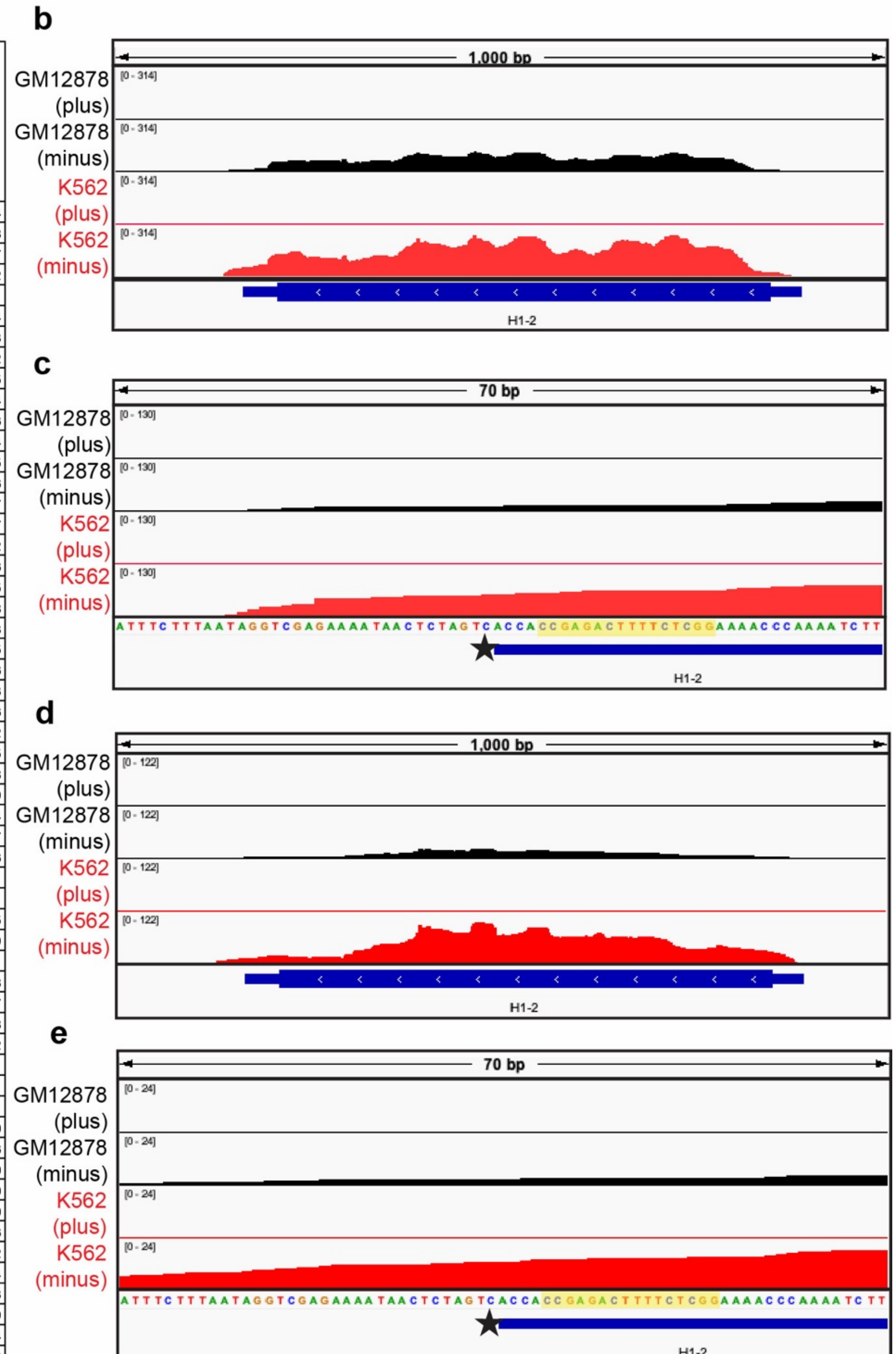

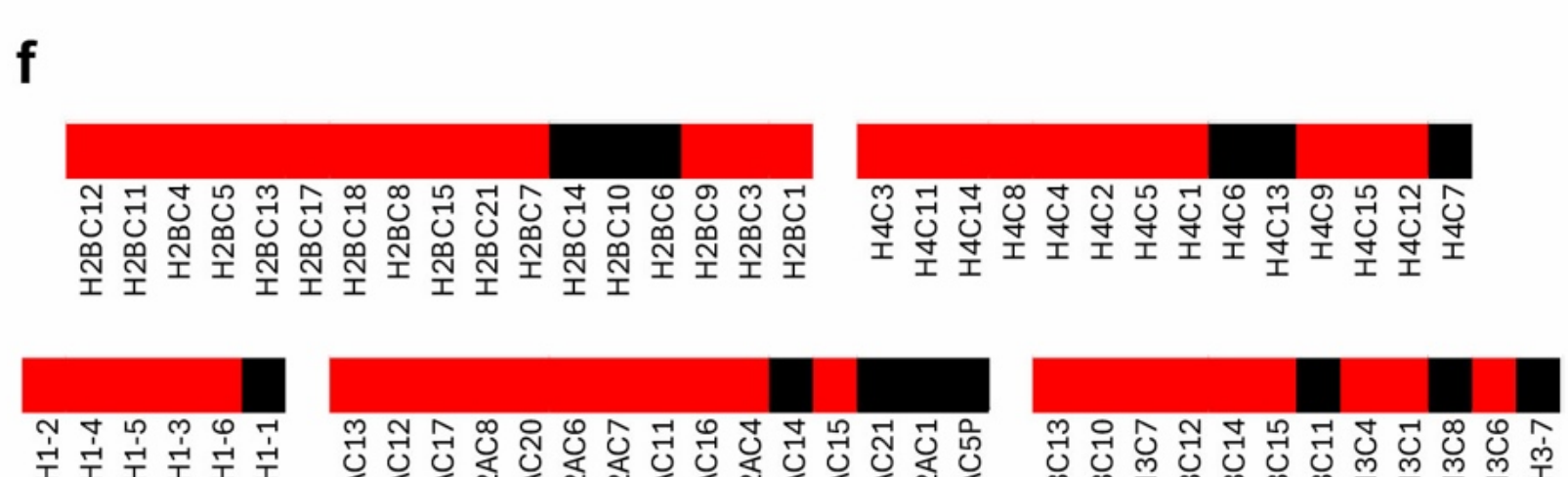

bioRxiv preprint doi: https://doi.org/10.64898/2026.10.14.758961; this version posted October 20, 2026. The copyright holder for this preprint (which was not certified by peer review) is the author/funder, who has granted bioRxiv a license to display the preprint in perpetuity. It is made available under a CC-BY 4.0 International license.

Supplemental Figure 5: Evidence for polyadenylated histone mRNA in ENCODE RNA-seq. (A) Table of histone gene expression in GM12878 and K562 cells by RNA-seq library type. Mean transcripts per million (TPM) for histone genes from ENCODE gene-level quantifications of poly(A)+ RNA-seq ("GM12878 poly(A)", "K562 poly(A)") and rRNA-depleted total RNA-seq ("GM12878 total RNA", "K562 total RNA") libraries (accessions in Supplementary Table 1). TPM was averaged across replicates within each experiment, and experiment means were then averaged within each cell line and library type. The difference in mean TPM between K562 and GM12878 for each library type were calculated ("poly(A): K562-GM12878" and "total: K562-GM12878"). Genes are ordered by largest to smallest difference in poly(A) libraries. **(B, C)** Strand-specific signal of uniquely mapped reads from rRNA-depleted total RNA-seq data at H1-2, the top-ranked gene in (A), for GM12878 (black) and K562 (red). (B) Coverage across the gene. (C) Coverage at the 3′ end, with the conserved stem-loop highlighted in yellow, and the canonical cleavage site located 5 nt downstream of the stem-loop marked by a star. (ENCODE accessions: ENCFF509KPQ, ENCFF045ITU, ENCFF312ZLI, ENCFF530FJG). **(D, E)** Same as (B, C) for poly(A)+-selected libraries. (ENCODE accessions: ENCFF243ECE, ENCFF763UJG, ENCFF536ZVM, ENCFF839ZOT). In both library types, signal extends beyond the canonical cleavage site on the transcribed strand, consistent with a polyadenylated H1-2 transcript. **(F)** Heatmap of histone genes for presence of a polyadenylated isoform in K562 cells. Genes were scored positive (red) if (1) signal over the coding sequence was present in both poly(A)+ and total RNA-seq, and (2) total RNA-seq signal extended downstream of the canonical cleavage site on the transcribed strand.

bioRxiv preprint doi: https://doi.org/10.64898/2026.10.14.758961; this version posted October 20, 2026. The copyright holder for this preprint (which was not certified by peer review) is the author/funder, who has granted bioRxiv a license to display the preprint in perpetuity. It is made available under a CC-BY 4.0 International license.

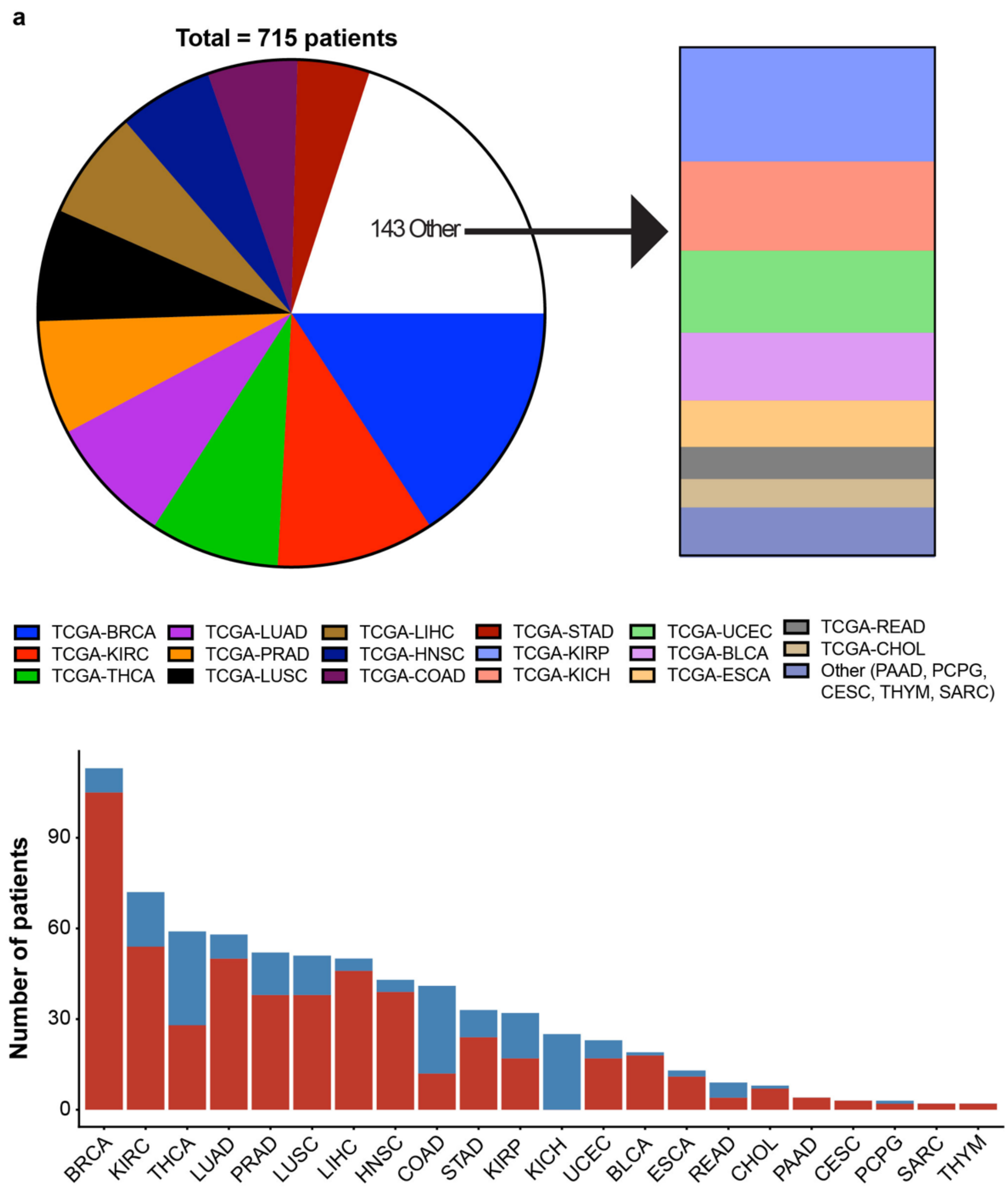

Supplemental Figure 6. Distribution of Matched TCGA Patient Samples. (A) Composition of the study population across 22 TCGA projects (n=715 total patients). Pie chart highlights the 10 most frequent project cohorts: TCGA-BRCA (n=113), TCGA-KIRC (n=72), TCGA-THCA (n=59), TCGA-LUAD (n=58), TCGA-PRAD (n=52), TCGA-LUSC (n=51), TCGA-LIHC (n=50), TCGA-HNSC (n=43), TCGA-COAD (n=41), and TCGA-STAD (n=33). Stacked bar chart describes 12 additional projects: TCGA-KIRP (n=32), TCGA-KICH (n=25), TCGA-UCEC (n=23), TCGA-BLCA (n=19), TCGA-ESCA (n=13), TCGA-READ (n=9), TCGA-CHOL (n=8), TCGA-PAAD (n=4), TCGA-PCPG (n=3), TCGA-CESC (n=3), TCGA-THYM (n=2), and TCGA-SARC (n=2). **(B)** Polyadenylated histone gene expression is upregulated in the majority of tumor samples across nearly all cancer types examined. Stacked bar plots show the number of patients with higher (red) or lower (blue) summed histone TPM in primary tumor relative to matched normal tissue, for each of 22 TCGA cancer types.

bioRxiv preprint doi: https://doi.org/10.64898/2026.10.14.758961; this version posted October 20, 2026. The copyright holder for this preprint (which was not certified by peer review) is the author/funder, who has granted bioRxiv a license to display the preprint in perpetuity. It is made available under a CC-BY 4.0 International license.

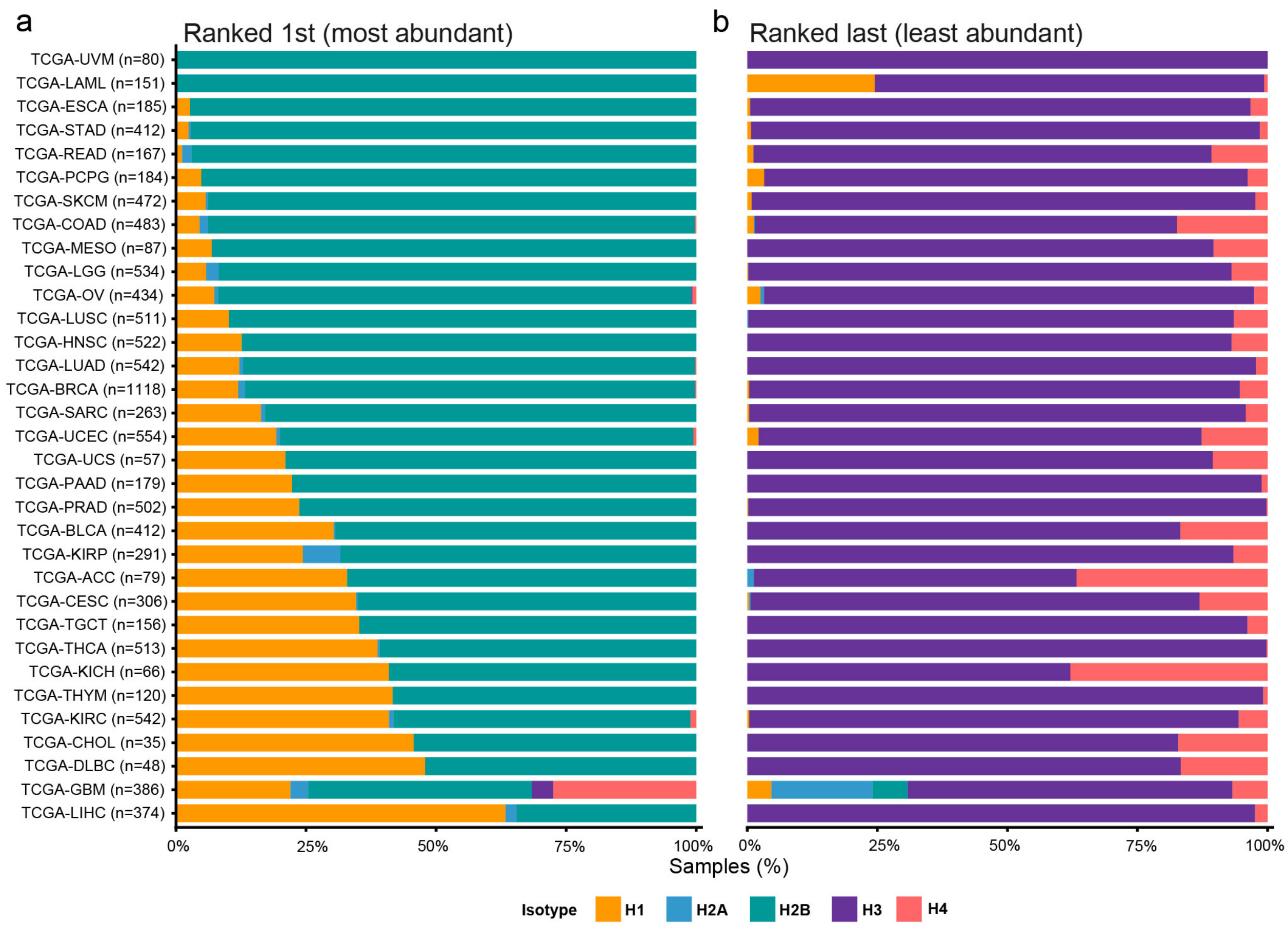

Supplemental Figure 7: Pan-cancer histone isotype rank frequency across 33 TCGA cancer types. (A, B) Stacked horizontal bar charts showing the frequency of histone isotype ranking among 10,765 RNA-seq samples across 33 TCGA cancer types. For each sample, relative abundance of each histone isotype was calculated and then ranked from most abundant (highest, ranked first) to least abundant (ranked last). The proportion of samples in which each isotype ranked (A) first and (B) last were quantified for each cancer. Cancer types are ordered by decreasing H2B rank frequency.

bioRxiv preprint doi: https://doi.org/10.64898/2026.10.14.758961; this version posted October 20, 2026. The copyright holder for this preprint (which was not certified by peer review) is the author/funder, who has granted bioRxiv a license to display the preprint in perpetuity. It is made available under a CC-BY 4.0 International license.

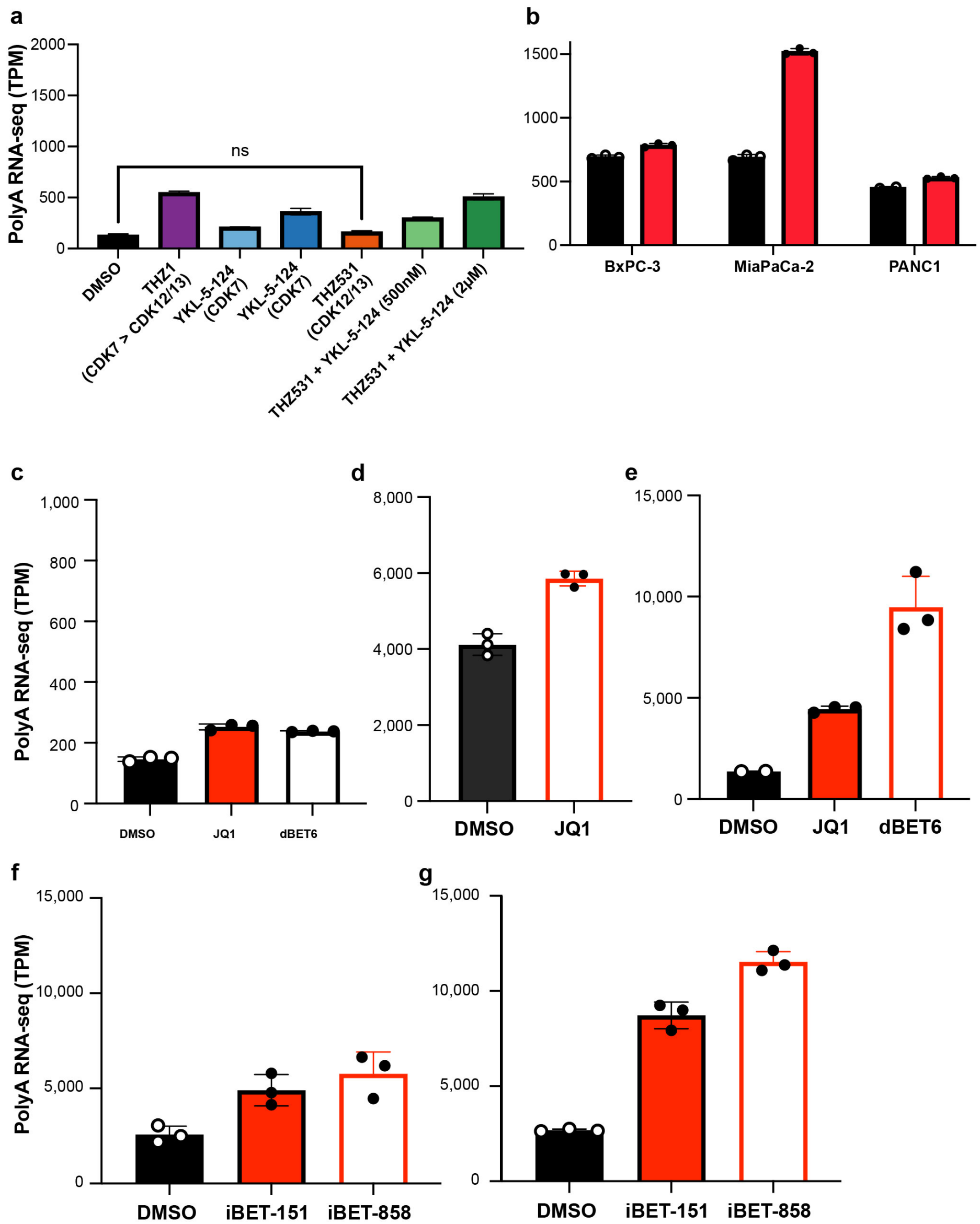

Supplemental Figure 8: Chemical and genetic dissection of polyadenylated histone gene regulation. (A) HAP1 cells expressing mutant CDK7-C312S treated for 24 hours with DMSO, YKL-5-124 (CDK7-selective), THZ1 (CDK7/12/13), THZ531 (CDK12/13-selective), or THZ531 combined with YKL-5-124 (21). One-way ANOVA with Dunnett's test; all comparisons significant (P adj $\leq$ 0.0007) except as noted (ns = not significant). **(B)** BxPC3, MiaPaCa-2 and PANC1 cells were treated with DMSO or THZ1 for 6hr (22). Unpaired t-test with Welch correction were run following Grubbs test comparing treated cells to control and were all found to be significantly greater in treatment (P $\leq$ 0.0031). **(C-E)** MOLT4 cells treated for 2h (C), T-ALL PDX for 6h (D), and primary T-ALL samples treated for 6h (E) with dBET6 or JQ1 (25). (C) One-way ANOVA with Dunnett's test; all comparisons significant (P adj < 0.0001). (D) Unpaired two-tailed T-test (P = 0.0009). (E) one-way ANOVA with Dunnett's test with all comparisons being statistically significant (P adj < 0.05). **(F-G)** OVCAR-3 ovarian cancer cells treated for (F) 4h and (G) 24h with either i-BET858 and i-BET151 (26). One-way ANOVA with Dunnett's test; all comparisons statistically significant (P adj < 0.05).
